# Being used for the greater good while fighting on the frontline: care staff’s experiences of working with older people during the COVID-19 pandemic in Sweden

**DOI:** 10.1186/s12877-023-04644-0

**Published:** 2024-02-06

**Authors:** Annica Lövenmark, Lena Marmstål Hammar

**Affiliations:** 1 The School of Health, Care and Social Welfare, Mälardalen University, Västerås, Sweden; 2 Division of Nursing, Department of Neurobiology, Care Sciences and Society, Karolinska Institute, Stockholm, Sweden; 3 The School of Health and Welfare, Dalarna University, Falun, Sweden

**Keywords:** Assistant nurse, Care aide, Geriatric care, Working conditions, Occupational health, Residential facilities, Home care service, COVID-19

## Abstract

**Bakground:**

Worldwide, older people were more severely affected during the COVID-19 pandemic than others. In Sweden, those living in residential care facilities had the highest mortality rate, followed by those receiving home care services. The Swedish and international literature on the working environment for assistant nurses and care aides during the pandemic shows an increase in stress, anxiety, depression and post-traumatic stress syndromes. Care organisations were badly prepared to prevent the virus from spreading and to protect the staff from stress. In order to be better prepared for possible future pandemics, the health and well-being of the staff, the care of older people and the experiences of the staff both during and after a pandemic are important aspects to take into account. Therefore, this study aims to describe the experiences of assistant nurses and care aides working in the care of older people during the COVID-19 pandemic in Sweden, their working conditions and the impact all this had on their lives.

**Methodology:**

The study has a qualitative, descriptive design. The data was collected in four focus group interviews with 21 participants and analysed using qualitative content analysis.

**Results:**

The results revealed the theme, *Being used for the greater good while fighting on the frontline*, which was then divided into three categories: *portrayed as a risk for older people, not being valued* and *being burnt out.* The worsening working conditions that the pandemic contributed to resulted in a high degree of stress and risk of burnout, with staff members both wanting to and actually leaving their employment. After the pandemic they felt forgotten again and left to cope in an even worse situation than before.

**Conclusions:**

The pandemic had a major effect on assistant nurses and care aides in terms of their working environment and their private lives. To be better prepared for future pandemics or disasters, organisations with responsibility for the care of older people will need to ensure that their staff have the necessary competencies and that there is adequate staffing in place. This also means that adequate government funding and multiple interventions will be needed.

## Background

 Around the globe, older people were more severely affected during the COVID-19 pandemic than others. In Sweden, those living in residential care facilities (RCF) had the highest mortality rate, followed by those receiving home care services (HCS) [1]. The government took the lead and coordinated the necessary strategies for combating the pandemic. By means of new regulations and recommendations, the provision of support and the necessary communications and advice, the government had a major impact on municipalities and regions. The strengths and weaknesses in management at all levels in the various care organisations were also revealed during the pandemic. In the care of older people, a major consequence was a lack of cooperation between municipalities and regions, which led to a reduced number or complete absence of doctors to evaluate older people’s needs [2, 3]. Also, RCF and HCS were generally unable to maintain strict hygiene and provide sufficient terminal care. Recent reports have also [2, 4] shown that organisations responsible for the care of older people in Sweden lacked the necessary hygienic routines and that their staff lacked competence in this area.

In addition, in the first months of the pandemic a lot of the necessary protective equipment was prioritised for hospital care, thereby leaving RCF and HCS badly prepared to prevent the virus from spreading. Also, the care of older people had a low test capacity, low staff continuity and the fact that the staff had to work even if they had mild symptoms of the virus [2, 5]. It also turned out that RFC had a higher proportion of hourly-paid staff that thus did not have sickness benefits when staying at home, and also had less staff before the pandemic [6].

As staff caring for older people in Sweden are mainly assistant nurses (AN) (with basic care education at upper secondary school level), or care aides (CA) (with no formal education) [7], their main assignments are to assist with everyday living, such as personal care and general housekeeping duties like cooking and doing the laundry. Medical measures are provided by registered nurses (RN), who only constitute around 5% of the staff. As they have overall responsibility for a large number of older people, they have to delegate medical tasks to ANs and CAs regardless of whether there is a pandemic or not. However, during the pandemic the roles of the staff changed rapidly to include duties that they were not prepared or qualified for, which included medical tasks related to health care measures and tasks related to preventing the spread of the virus, both of which were more advanced than ANs and CAs had experience of or were trained for [5, 8]. Especially in HCS, staff commonly work alone and need to rely on their own skills in more advanced tasks than they are trained for [9]. In addition to being afraid of spreading the virus to older people, they were also concerned about their families’ and their own emotional and mental well-being [10, 11].

Both the Swedish and international literature on the work environment during the pandemic shows an increase in stress and anxiety as well as depression and post-traumatic stress syndromes in the nursing and care home context [12–16]. There are several reasons for this. Some are related to the poor working conditions and capacities in the care of older people even before the pandemic. This made organisations badly prepared to prevent the virus from spreading, but also to prevent stress syndromes amongst the staff [8, 17–19]. Also, the high number of deaths over a short space of time and families not being able to be with their loved ones led to moral distress [20, 21]. The managers commonly worked from at home in an attempt to prevent the spread of the virus [2], which made the staff feel abandoned [22] and undervalued. They were also accused by the residents’ families and the mass media of having spread the virus and providing low quality care [18, 20, 21].

Registered nurses employed in the municipal care sector in Sweden have the highest medical competence in the care of older people and are meant to supervise ANs and CAs. During the pandemic, also their work situation was severely strained, which resulted in inadequate communication and cooperation with staff and management [3, 23]. The staff in turn found it difficult to understand and follow the guidelines for preventing the spread of the virus without supervision, as the guidelines were often changed at a moment’s notice, which made it difficult to convert them into practice [5, 22]. Other studies have found that the implementation of the guidelines was ineffective [24] and lacked robust planning [25]. At the same time, a Swedish study showed that ANs and CAs felt that no-one was listening to their experiences and views, even when they wanted to contribute to the development routines for the pandemic [20]. The fact that ANs and CAs felt undervalued, marginalised and excluded from involvement in care decisions was also highlighted in research prior to the pandemic [26, 27].

Even before the pandemic the care of older people in Sweden was strained, mainly due to organisational and regulation changes over the last decades and more older people needing care. As the number of staff has not increased to match this growth, it has led to high staff turnovers and short-term and hourly paid appointments [7]. Previous research has revealed an increase in workloads, stress and burnout [28–32]. A recent national report [33] has shown that in 2023, 60% of ANs and CAs, compared to 45% in 2017, are willing to leave their work of caring for older people due to the psychological and physical demands, not being able to provide satisfactory care, a lack of support from managers, not being able to combine working and private life, time pressures, low salaries, unsocial working hours and being unable to influence their work situation. In a study comparing the situation in 2005 and 2015, it was found that in 2015 there were larger numbers of care recipients, that staff received less training, experienced less support from managers and were unable to plan their own work [28], which impacted both their well-being as individuals and their organisational work environment [28, 34–36]. Not surprisingly, a study of HCS staff found that job strain was correlated to not feeling in control at work, a low organisational culture and climate and a weak leadership [37]. The literature on the working environment has highlighted that experiencing high job demands, working independently without any appropriate support, feeling out of control, or being unable to manage issues during work, are associated with high levels of job strain [38], and that balancing empowerment, organisational structures, good management and a psychological work environment are essential parts of a supportive working environment [39–41].

Even before the pandemic, the organisation of how older people were cared for in Sweden was strained. During the pandemic, ANs and CAs were obliged to perform tasks that they did not have the skills or training for, or the prerequisites for them. They were also at the frontline working in close contact with older people and were accused of not being able to save lives and/or provide sufficient care. In order to be better prepared for possible future pandemics or other disasters, and to ensure the health and well-being of care staff, as well as the care of older people, the experiences of ANs and CAs during the COVID-19 pandemic are important to take into consideration. Thus, the study aims to describe the experiences of assistant nurses and care aides working in the care of older people during the COVID-19 pandemic in Sweden, their working conditions and the impact all this had on their lives.

## Methods

### Study design

The study has a qualitative descriptive design and uses focus group interviews (FGIs) [42] to gain a deeper understanding of the working experiences of ANs and CAs during the COVID-19 pandemic.

### Setting and sample

The people taking part in this study were assistant nurses (ANs) working in municipal and private RCF for older people or in HCS in Sweden during the COVID-19 pandemic. Four of the managers in these RCFs and HCRs were registered nurses, seven were social workers, five were assistant nurses, one was a physiotherapist, two were behaviour scientists and two were economists. In the municipal care of older people, ANs and CAs main tasks are assisting patients in their everyday living, including personal care, housekeeping, laundry and cooking regulated by the Social Service Act [43]. In RCF, the staff mostly consists of ANs and CAs working together with the residents. In most RCF, RNs are employed to focus on health care measures regulated by the Health Care Act [44] and are mostly not involved in the everyday care of the residents. RNs commonly have responsibility for more than one residential care facility. In HCS, ANs and CAs often work alone in older people’s ordinary homes, while RNs are more medical consultants. Governed by the Social Service Act [43], the main priority in the municipal care of older people is to increase and support their everyday lives. Health care professionals are few and no doctors are directly employed. The health care professionals with the highest medical competence are RNs. In contrast, the health care system in hospitals is legalised by the Health Care Act [44], where doctors are employed and where a large number of RNs and ANs focus mostly on curative care and the Swedish population at large.

Professions other than ANs and CAs that were not involved in the everyday care of older people were excluded, e.g. administrative staff or registered nurses. Most of the participants were recruited through trade unions and a few by Facebook groups for people working in RCF and HCS. Based on a Swedish survey of the working life and conditions of ANs and CAs during the COVID-19 pandemic with 1 114 people from different parts of Sweden, 232 noted their contact details in order to volunteer for FGIs as a follow-up to the survey. All these 232 people were contacted during the spring of 2022. Several and repeated attempts were made to form at least four focus groups: two from RCF and two from HCS, with four to six participants in each group. The participants in each group came from different workplaces in Sweden.

### Data collection

In April and May 2022, data was collected from four FGIs [42] with three groups from RCF and one group from HCS, including 12 ANs and three CAs from RCF and five ANs and one CA from HCS: a total of 21 participants. Three groups consisted of four participants, one group of three participants (RCF) and one group had six participants (HCS). All the participants except one were female and four had worked in the care of older people for 4–7 years, while the other participants had working experience of between 27 and 40 years. The majority of ANs worked partly as assigned union representatives. The interviews were conducted by Zoom and the sound only was recorded using dictaphones. The interviews consisted of open-ended questions and were guided by the following: Please tell us about your work, working conditions and working environment, your health, your managers and instructions and abilities to prevent the COVID-19 virus during the pandemic. The first author was the moderator and assistant in two interviews and the second author was the moderator and assistant in two interviews. Both authors have solid and long-standing experiences of qualitative research and different kinds of interviews as ways of collecting data. There were no previous relationships between the authors and any of the participants. One interview lasted for 55 min, one for 97 min, one for 104 min and one for 110 min.

### Data analysis

The data was analysed using manifest qualitative content analysis [45] and transcribed verbatim. The text was read through several times to gain a full understanding of it. Next, parts of the text, such as sentences or meaning units related to the aim of the study, were identified and coded using NVivo (see Table 1). Meaning units focusing on the working experiences of staff in HCS and RCF were marked and assigned to separate groups based on the similarities and differences between them. This was followed by condensing the meaning units and abstracting them into codes. Theses codes reflected the aim of the study regarding the working conditions connected to the text and context [45]. The codes were then analysed for their similarities and differences. By going back and forth in the analytical process, the analysis resulted in a detailed and overall understanding of the content. This process also included an abstraction of the codes into six sub-categories, three categories and one main theme (see Table 2). Both the process and the results of the analysis were discussed several times in order to reach consensus.

**Table 1:** Example of the analysis process

Meaning units	Condensed meaning unit	Codes	Sub- category	Category	Theme
It’s only the region that has been provided with cakes, energy drinks, sweets, biscuits, sandwiches etc. just because they are so much better at it. But we haven’t received anything at all	Those in the region have been given cakes etc. but we got nothing	No credit	Being left out	Not being valued	Being used for the greater good while fighting on the frontline
We were expected to do everything they said and if we weren’t able to or didn’t manage to do it they didn’t listen	Just do what they said, and they didn’t listen if we were unable or didn’t manage to do it	Work harder without being seen	Taken for granted by the managers

**Table 2:** The result presented in six sub-categories, three categories and one theme

Sub-category	Category	Theme
Designated by relatives as those who infected the older people Designated by the media as spreading the virus Being left out Designated by the managers as a high risk Not being able to cope Not getting any recovery time	Portrayed as a risk for older people Not being valued Being burnt out	Being used for the greater good while fighting on the frontline

### Ethical considerations

The study was approved by the Swedish Ethical Review Authority and allocated the register number 2021 − 00486. Participants were informed by the authors both orally and in writing about the aim of the study and the research ethics: for example, the voluntary nature of participation. The ethical standards for scientific work were followed and based on the Declaration of Helsinki [46].

## Results

The overall theme, “Being used for the greater good while fighting on the frontline” included the following three categories: “Portrayed as a risk for older people”, “Not being valued” and “Being burnt out”. The results showed that the staff experienced that the older people’s relatives and the media designated them as a risk in terms of older people being infected by the virus. The staff did not experience that they were valued in the same way as their counterparts in other professional contexts, such as in hospitals, and they often felt taken for granted by their own managers. Their challenging working conditions both during and after the most intense period of the pandemic meant that they found it difficult to cope at work and were not given any recovery time.

### Portrayed as a risk for older people

The category “Portrayed as a risk for older people” consists of two sub-categories: “Designated by relatives as those infecting the older people” and “Designated by the media as those spreading the virus”.

### Designated by relatives as those infecting the older people

All the participants from RCF and HCS expressed that they were designated by the people around them as bringing the virus into older people’s own homes or residential care homes. When the older people’s relatives visited, they were not regarded as the same risk. When the staff tried to follow the instructions about protective equipment and that relatives could visit without this equipment, they were still designated as spreading the virus. It is described as an ironic and contradictory fact that the spread of the virus could not have happened when relatives embraced an older person or moved around in society:

… it is only we staff who can give them corona (HCS, RCF 1–3) …. and not the relatives who can come in and hug their relatives without face masks – who can give them corona … not even when they move around in society … (RCF 3)…//… it’s as if it’s only us, the staff, who can give them corona (HCS, RCF 1–3).

Several of the staff were infected with the virus at work but were not allowed to say so. They were expected to align with the widespread and “known fact” that it was always the staff who brought the virus into older people’s homes and infected the residents. It could not possibly be the relatives or other people who visited them. For some of the staff this was the hardest thing to cope with, and still is, even when the pandemic is no longer considered a threat to society:

…I’m 100% sure that I was infected at work and by whom, but I wasn’t allowed to say anything because it was always reported as being the staff who had introduced the virus to all the residents and that it couldn’t have been the relatives or anyone else who were out and about. It was always the carers and nursing staff who had introduced the virus everywhere. And that has been hard, although I almost think that it has been harder to bear afterwards… (RCF 2).

### Designated by the media as those spreading the virus

All the interviewed staff described how the news media accused them of spreading the virus to older people’s homes by naming them as “those who kill” (RCF 2, HCS) and “those who infect” (RCF 1–3, HCS). Several of them described it as a witch-hunt and that the news media painted an overall negative picture of both RCF and HCS: “…a witch-hunt of assistant nurses in the newspapers as well”: “The staff took Covid into the home, five died … that was extreme…” (RCF 1, 3, HCS). One of the participants from an HCS described how the news media wrote a lot of negative things about them, stated that HCS did not function and compared it with private care that worked well. The staff described it as always being in some way allowed to speak disparagingly about them and their work. They wished that had politicians come to see the work and their working conditions for themselves:

Well there was a lot of negativity about the home-help service… that it didn’t work, about the staff… they didn’t have time…weren’t able to do what they were supposed to do etc. …//… how much better it was in the private sector…it feels a bit as though the home-help service is the one everyone throws mud at… all the time…//… the politicians should have come and worked with us to see the reality… it wouldn’t have hurt (HCR).

The people that the staff met outside work were influenced by the news media designating them as the main risk to older people, especially at the beginning of the pandemic. The news media’s reporting was commonly regarded as factual by the staff’s family and friends:

…my friends and family repeated the ´facts´ that news media published - us being the ones spreading the virus to the most vulnerable…” (HCR)…//…it was tiresome that people around you just took the media as knowing everything and who to blame for spreading the virus (RCF 1)…//… at the beginning everybody around you only listened to the media and thought that we were a greater risk for older people… (RCF 2, 3).

One of the staff described the meeting with the staff at a pizzeria after working for 15 h:

I’m so sad… even after a 15-hour stint…I buy pizza on the way home…I’m too tired to cook…and then even from the guy at the pizzeria…but he had heard it on the TV and had seen the awful things (that they say we do)? (RCF 1)

In the above quote, the staff member describes the stress at being faced with the news media’s reports about how bad the NAs and CAs were and the level of danger they exposed the older people to.

The category,” Not being valued” consists of the sub-categories “Being left out” and “Designated as a high risk by the managers”.

### Being left out

During the pandemic, RCF and HCS staff noted that other parts of the health care service also received a lot of media attention, but in contrast were praised by people in society. The care and nursing staff in e.g., hospitals had food, money, drinks, sweets and cakes sent to them as signs of appreciation. However, the staff in the home care residential services received nothing at all:

The home-help services have not been prioritised in the same way as others (HCR, RCF 1–3) …the accident and emergency units have been told… ’yes, but you are fantastic’, people have swished lunch money and businesses or restaurants have delivered lunches…//…we haven’t seen anything of the sort…//…no businesses have called us fantastic. Only the region has got cakes, crates of energy drinks, sweets, biscuits, cakes and sandwiches… only because they are regarded as fantastic, but we haven’t got anything (RCF 1). We did not get lunches and snacks like those at the hospitals (HCR, RCF 2).

Both during the pandemic and afterwards the staff experienced that people in general did not remember all their hard work. When all the restrictions were over and people went back to their ordinary lives, they forgot all about them and the other professionals who had tried to fix things in the best way possible. They felt forgotten and unnoticed:

…but people have forgotten how much we did…they have forgotten all the restrictions now…//…now we can live again…but they have forgotten how many staff…cleaners, caretakers…people who delivered food…everyone…not only the assistant nurses and nurses but everyone who mucked in and tried to fix things, people have forgotten all those (RCF 2). Now they have forgotten all our hard work and everything we had to do… (RCF 1–3, HCR) …//… it´s as if they don’t see you anymore (RCF 3).

During the pandemic the staff were not noticed and even now, after the pandemic, they were no longer seen as important, no-one remembered them, and it was no longer significant that they protected themselves or protected the older persons. They were simply left to cope on their own: “you’ll just have to manage the best you can now…//…we are no longer important…people have forgotten now” (RCF 3).

### Designated by the managers as being a high risk

Two of the staff from residential care facilities described that they were well supported by their managers: “ the boss has been there and joined in the work when needed” (RCF 2)…//…they had full control of the situation and understood our frustration and so on (RCF 1). However, the majority of staff described that they did not feel listened to, felt used and had to work very hard. Some of the staff had to do the manager’s work and inform those older people who did not get any help on a particular day that there was no time.

…you’re not a person, just someone who should do everything and work harder and harder (RCF 1) …//…we were expected to do everything they said and if we were unable or didn’t manage to do it they didn’t listen and we had to inform those who couldn’t get any help ourselves (HCR)…//… we were just supposed to do what we’d been asked to do and not ask any questions (RCF 3).

When talking to the manager about the heavy workload, this particular staff member was advised to look for another job. As there was a huge shortage of staff, the staff who were available “are on their knees” due to the constant and heavy workload:

…you have to work yourself to death and are not given any credit or anything (when you protest), they just say that you can look for other employment…it’s the worst thing you can say to anyone, really…there’s hardly any staff and those who remain are on their knees…so they’ve had to work all the time… (RCF 2).

The staff described that the managers played on the staff’s consciences regarding their colleagues’ work situation in order to get them to work more and harder. The managers themselves stated that they did not have time to do things and the staff’s working environment was not something they could take into consideration or prioritise: “they rely on my conscience, they know that our colleagues have to work all hours…while the managers complain that they don’t have time and can’t prioritise the working environment” (RCF 3).

The staff’s consciences regarding the older people and their need for human contact and care were also made use of by the manager: “they know that we’ll do anything for the older persons and it’s clear that they take advantage of that” (RCF 1) and that “they know that the older persons only have us and that we’ll do everything we can” (HCR). The result was frustration and loneliness, which the staff had to deal with themselves. Their working conditions during the pandemic included being more than just caregivers, in that they also tried to replace the older people’s lack of contact with others.

… their frustration and loneliness…they just have to bear it in some way…and also that we’ve had to be more than nursing and care staff…we’ve had to take on many different roles…when they know that two colleagues and I are the only human contact, apart from telephone calls, that they’ve had all day (HCR).

The category “Being burnt out” consists of the sub-categories “Not being able to cope” and “Not getting any recovery time”.

### Not being able to cope

All the staff shared the experience of no longer being able to cope and about a lot of colleagues resigning or who were about to do so. All the hard work during the pandemic had caught up with them to the extent that they were no longer able to cope or continue working, regardless of whether they had another job to go to or not:

People are leaving now because they can no longer cope… (RCF 1–3, HCR) …//… it’s as if they’ve reached rock bottom…it’s too much…people are leaving and finding new employment, some have jobs to go to and some have nothing, they just feel empty… (RCF 1). Those who can take early retirement… (RCF 2, HCR).

The staff tried hard to make their voices heard by those in charge, but did not seem to have been listened to, which made it difficult to continue working. The staff who were still at work said that they did not know whether they would be able to take any holiday time at all the following summer due to the shortage of staff: “it’s tough, we have a huge shortage of staff…there’s a lack of 14 summer interns…there simply isn’t the staff…we can’t go on holiday…it’s tough” (HCR).

All the hard work undertaken during the pandemic came on top of an already heavy workload, but that had to be forgotten and all the stops pulled out to deal with the pandemic and its effects. When the pandemic receded all the old problems reappeared, such as conflicts and having to deal with new staff. All this was very difficult to cope with. One thing that changed both during and after the pandemic was that the engaged staff who were unable to cope anymore left. That was critical, because they had their “hearts in the right place” and worked in the care of older people care for the “right reasons. They were unable to stay and preferred to retire if they could in order to have an ordinary social life:

…it’s the engaged staff who leave…(RCF 1–3, HCR)…//… those with their hearts in the right places…(RCF 2)…//…we who go to work for the right reasons and care about the people who live there, it’s us that can’t cope…//…I’ve worked here for 30 years and have just sent in my resignation letter because I feel that it’s time to retire…I also want to have time for my grandchildren and so on…and I don’t now… (RCF 2).

### Not getting any recovery time

When the work and workload was too difficult the opportunity to recover became even more crucial. The staff said that they lacked both the time and the possibility to recover and simply wanted some leisure time instead of having to deal with an aching body and head, so that they could think and not need to go to bed straight away when they came home. After work they were very tired and lacked the energy to do the things they were capable of before the pandemic:

… when I come home from work I want to feel that my body and head do not ache…that I can think…when I come home now I just want to go to bed…I’m so tired, very tired, but before I was able to do lots of things when I came home…I can’t now, I’m completely worn out… (HCR) …//…you don’t have the energy for more than work and hardly even that (RCF 1)…//… there’s no energy left when you come home… (RCF 2).

Before the staff were able to enjoy their leisure time but now there was a lack of drive and desire to do anything when their energy levels were so low. The distressing work situations during the pandemic also meant that they were unable to sleep and were in a continuous state of anxiety: “those awful situations, they really made you anxious…//…there’s no energy left… (RCF 3).

Even when it was possible to rest, the staff described the situation as being impossible to cope with. It was no longer possible to have a social life because they were much more tired than they were before the pandemic. Coping was much harder now:

…and you can’t even manage to be social in your time off because you’re so tired…and everyone who worked during the pandemic says the same…that they are much, much more exhausted now…you feel as though you don’t get the recovery time you need…even if you do have time off during the week it’s difficult to recover…it’s much tougher now (RCF 2)…//… it was tough before the pandemic but then, then it just got worse and worse and now, now we’re even more tired (RCF 1)…//… there are many now who simply don’t have the energy for more, it’s worse now… (HCR).

## Discussion

The results reveal that an already strained working environment in the care of older people became almost unbearable during the pandemic. Staff experienced being used for the greater good while fighting on the frontline to protect the older people. As confirmed by previous research and reports [2, 5], they had to do things that they were not prepared or qualified for and at the same time experienced not being supported by the managers or RNs in their organisations and even being accused of causing the spread of the virus. In the literature on work related stress, being supported by managers and being competent enough to undertake work tasks are crucial for a healthy working environment [47, 48]. Previous research on job strain in RCF revealed that a supportive leadership was highly correlated with lesser job strain. A report (also in press) of RNs working conditions [3] in the care of older people during the pandemic also found that perceptions of support were correlated with the education of the managers, revealing that those with managers with medical competence (mainly RNs) experienced more support than those managers without. Only four of the managers of the participants in our study were RNs and had medical competence, so it could be said that some of them may have lacked competence in how to minimise the risks of spreading the virus and increasing the care of those infected, and that they thus found it more difficult to support the staff’s efforts. A lack of management support has been reported in previous research [20, 21] and can be seen as a major failure, especially in extraordinary situations like a pandemic, where leadership is essential. Previous research has further shown that leadership is a crucial factor when it comes to minimising work strain in general [40, 41] and is even more important during a pandemic [34, 49]. It should also be recognised that care managers in Sweden oversee large numbers of staff (on average 42 staff members in home care and 50 in RCF), which means that providing support for each member of staff is challenging [50] and could be even more so during a pandemic.

The results also show that the participants felt pressure from their managers to work harder, which gave them bad consciences about not having enough time to spend with the older people who needed their contact even more due to the absence of relatives. This has also been highlighted in research [51] before, especially in the care of older people, as a “stress of conscience”, which associates with negative outcomes such as burnout, moral burdens, workplace stress and the inability to offer a high quality of care.

Our study also corroborated what previous studies have revealed [18, 20], namely that the media positioned them as lacking competence and causing the contamination of older people, in comparison with how the media reported hospital care, and especially intensive care units, which were highly appreciated and positively presented [52]. Those workers were described as a source of strength and as a reason for continuing to work [53], whereas the staff caring for older people felt left out with no chance of explaining their situation or being involved in developing it [20]. The problem is complex, but with regard to society and the media, it raises questions about the public’s knowledge of the care of older people and the influence and responsibility of the mass media reporting on it. Possibly, the relatives’ worries (and the staff’s) were increased by the reports in the mass media, which led them to easily designate the staff as spreading the virus and contaminating older people, as found in the study conducted by Bergqvist et al. [20]. Another study showed that the role of the media was unprecedented during the pandemic, with the publication of misleading information that led to distrust. The media may also have played a significant role for the staff’s experiences, and not only the factors related to their direct work situation [22]. This was also found to be the case in a study of care managers during the pandemic, which showed that they experienced the media coverage to be negative and incomplete, causing feelings of sadness and shame amongst themselves and their staff. This in turn generated an added workload, as they had to arrange for discussions to answer questions from the staff and the residents’ close relatives [54]. The Corona Commission further revealed that politicians and other governmental agencies did not understand how the care of older people was organised, and that the deficiencies that became apparent, such as the shortage of staff, the education of staff and lack of health care staff, primarily RNs, were reasons why the pandemic had a negative effect on the care of older people [5]. Lethin et al. [22] suggested that the media may even have influenced the politicians in this matter. It could also be the other way around, namely that the media publications reflected the dominant political views [55].

The stressful situation described by the ANs and NAs during the pandemic is extraordinary, although as previous research has shown, during the last decade the organisation of the care of older people has changed and the work environment has deteriorated [28, 33], which has led to staff resigning in greater numbers [30]. The pandemic naturally made the situation worse, as is clear from our results [20, 22]. However, during the pandemic the participants described not being able to cope at work or during their leisure time because they were too exhausted to exercise or be social with the people around them. This is alarming, since coping and the ability to recover are of the utmost importance for a person’s well-being. The question is whether they will be able to recover now that the pandemic is no longer regarded as a threat to society, given that care organisations are already strained and lack staff. This, along with other factors described previously, may be why staff continue to leave or want to leave their work [33].

In sum, we know that the care, safety and well-being of older people was affected during the pandemic [56–58] for several reasons [5, 59]. For decades, research has pointed to the need for improvements and suggestions, but it was not until the pandemic arrived that those needs became obvious [5]. In Sweden, the Corona Commission [5] and the Health and Social Care Inspectorate (IVO) [60] stated that older people in residential care and HCS were in need of advanced care, which required medical competence regardless of the pandemic. They also raised the question of inadequate staffing affecting the staff’s health and well-being and the safety and well-being of the older people in their care. IVO [60] further suggested that the lack of RNs and insufficient access to physicians resulted in ANs and CAs, with little or no adequate education, being left to carry out medical tasks that they were not qualified for.

### Strengths and limitations

One limitation of this study was the difficulty in finding participants, especially from HCS, to take part in focus group interviews. Many of the possible participants who had noted that they wanted to be contacted replied that they had either left their employment, were on sick leave, or did not have the energy to participate even if they thought it was important. It was therefore not possible to compare the staff’s experiences in these different care services. However, a strength was that the focus group interviews with the staff in both HCS and RCS could be conducted and that the results show similar experiences for all the staff regardless of their educational level or workplace. The purpose of the analysis was to describe the experiences and working conditions of ANs and CAs, which is why we chose to conduct a manifest content analysis as described by Graneheim and Lundman [45]. However, if aiming for more in-depth experiences, in-depth individual interviews and a latent analysis may be preferred. The steps of the analysis were helpful in moving between the transcripts, codes, sub-categories, categories and the theme, thereby making sure that the interpretations of the participants’ experiences were correct. We also discussed our findings with the staff caring for older people to confirm our findings.

### Conclusion and implications

The result of this study highlights the already known shortcomings in the Swedish welfare system regarding care for older people. In contrast to reports about the lack of care for older people and the high number of deaths during the pandemic, the staff’s own experiences during and after the pandemic are here brought to light in a way that stresses the importance of change in the future, regardless of whether there is a new pandemic or not. According to the participants, the pandemic brought them, their working conditions and this part of the caring system to a tipping point, which the government and the media should no longer ignore. To be better prepared for future pandemics or disasters, the voices and experiences of ANs and CAs need to be heard when standing on the frontline trying to protect older people. Organisations of care of older people also need to be developed to ensure adequate staffing, competencies and leadership. To enabling this, more government funding and multiple interventions will be needed.

## Data Availability

The data presented in this study is available on request from the corresponding author. The data is not publicly available due to restrictions in accordance with the Swedish Public Access to Information and Secrecy Act (2009:400). Requests for access to the dataset should be sent to the corresponding author and will be considered by the university’s data protection officer.

## Abbreviations

ANs: Assistant nurses (AN) have received basic care education at upper secondary school level Upper secondary level or care aides (CA) have no adequate formal education
COVID-19: Coronavirus disease 2019
HCS: Home care service
RCF: Residential care facility
